# Two Urinary Peptides Associated Closely With Type 2 Diabetes Mellitus

**DOI:** 10.1371/journal.pone.0122950

**Published:** 2015-04-22

**Authors:** Man Zhang, Guangzhen Fu, Ting Lei

**Affiliations:** 1 Department of Clinical Laboratory, Peking University Ninth School of Clinical Medicine, Beijing Shijitan Hospital, Beijing, China; 2 Department of Clinical Laboratory, Beijing Shijitan Hospital, Capital Medical University, Beijing, China; Moffitt Cancer Center, UNITED STATES

## Abstract

**Objective:**

To monitor of type 2 diabetes more simply, conveniently and noninvasively, we are trying to identify the potential urinary peptides that associated with different stages of glucose control in type 2 diabetes mellitus.

**Methods:**

Firstly, we collected urine samples from type 2 diabetic patients and normal controls. These type 2 diabetic patients were divided into two groups according to fasting plasma glucose (FPG) and hemoglobin A1c% (HbA1c), respectively. Magnetic beads based weak cation exchange chromatography (MB-WCX) was used to condense urinary peptides. The eluates were then analyzed by matrix-assisted laser desorption/ionization time-of-flight mass spectrometry (MALDI-TOF-MS). Subsequently, ClinProt was used to profile and screen the polypeptide patterns based on different methods of grouping in diabetic patients and normal controls. Finally, the amino acid sequences of differentially expressed peptides were identified by nano-liquid chromatography-tandem mass spectrometry and the protein sources of the corresponding peptide were matched in IPI Human database.

**Results:**

Proteomics analysis found two up-regulated peptide (m/z 2756.1 and m/z 3223.2) representations in diabetic subjects, and the two peptides increased with increases in the amount of glycosylated hemoglobin. Further, the parallelism between m/z 3223.2 and glycosylated hemoglobin was better than the parallelism between m/z 2756.1 and glycosylated hemoglobin. Area under the receiver operating characteristic of the two peptides was 0.722 and 0.661, respectively. The above-mentioned peptide m/z 2756.1 was further identified as fragment of fibrinogen alpha chain precursor and m/z 3223.2 was fragment of prothrombin precursor.

**Conclusion:**

These results suggested the two urinary biomarkers enable monitor of type 2 diabetes patients with different stages of glucose control.

## Introduction

Diabetes mellitus is a systemic metabolic disease characterized by hyperglycemia with insulin resistance and beta cell dysfunction. The International Federation of Diabetes published the Diabetes Atlas on November 14, 2013 which showed the number of the diabetes population had been enlarged in both developed countries and developing countries, and worryingly, the age of onset was falling [1]. It was estimated that as many as 30% and up to 90% (data is diverse from country to country) of type 2 diabetes patients remained undiagnosed and often the onset of diabetes occurred 4–7 years or more before diagnosis [2, 3]. The clinical diagnosis and glucose intolerance evaluation rely on the measurement of glucose level: random fasting plasma glucose test (FPG), oral glucose tolerance test and hemoglobin A1c (HbA1c). However, random fasting plasma glucose test requires an overnight fast, has high variability and lacks sensitivity. Oral glucose tolerance test is inconvenient and has weak reproducibility. Hemoglobin is the reflection of the glucose metabolism in the past six to eight weeks and cannot be used to diagnose diabetes. On the other hand, measurement of hemoglobin A1c needs lysed erythrocytes and thus adds extra work for laboratory staff.

Considering the increasing global prevalence of diabetes, especially under the hyperglycemic state, it compounds risk for life-threatening micro and macrovascular complications [4]. Moreover, it has also been associated with the increase in albuminuria [5]. So, it’s imperative to control strictly of glucose for diabetes patients though the use of a more convenient, reliable test. However, there still is not enough evidence to systematically expounded pathobiologial mechanism for diabetes under the current state of research.

Proteomics is a useful tool in the post-genomics era, offering the possibility to investigate and analyze all the proteins simultaneously in a particular sample [6]. State-of–art proteomic technologies enable analysis of differential protein expression in patients versus normal controls. These approaches have been applied to a variety of clinical specimens such as serum or plasma [7], kidney tissues [8] and urine [9] to look for biomarkers of diabetes mellitus. Among these, urinary proteomics has been widely used in basic and clinical research [9–11] with its unique advantages. Like other body fluids, urine contains a multitude of peptides and proteins that undergo disease specific modifications.

Our research will take advantage of bead based matrix-assisted laser desorption ionization time-of-flight mass spectrometry (MALDI-TOF MS) unveiling the differently expressed urinary peptides that are associated with different stages of glucose control for type 2 diabetes mellitus.

## Materials and Methods

### Study subjects

Firstly, the ethics committee of Beijing Shijitan Hospital, Capital Medical University approved the research project. Then, patients with type 2 diabetes mellitus and normal controls were recruited from Beijing Shijitan Hospital from February 2013 until June 2013. The participants all gave written informed consent, which was in accordance with the provisions of the Helsinki Declaration and approved by the ethics committee of Beijing Shijitan Hospital, Capital Medical University. Individuals with type 2 diabetes, defined as the use of oral glucose-lowering treatment or a fasting plasma glucose >7.0mmol/l (126 mg/dl) or nonfasting plasma glucose >11.1mmol/l (>200 mg/dl), were eligible for this study. Normal controls were chosen after a clinical check of renal function, blood pressure, microalbuminuria and urinary sediment. Finally, urine samples were collected from 29 normal controls and 49 patients with type 2 diabetes.

These forty-nine type 2 diabetic patients were divided into two groups according to the level of FPG and HbA1c, respectively. Firstly, grouping type 2 diabetes mellitus patients based on the level of FPG. FPG greater than 7.0mmol/l and less than 8.5mmol/l was defined as GM1 and that greater than 8.5mmol/l was defined as GM2. Then, grouping type 2 diabetes mellitus patients based on the level of HbA1c%. HbA1c% greater than 6.0 and less than 7.5 was defined as BM1 and that greater than 7.5 was defined as BM2. N represents normal controls.

The clinical characteristics of normal controls and type 2 diabetes patients are shown in Table 1 and there was no significant difference in sex, total cholesterol level, LDL cholesterol level, HDL cholesterol level, triglyceride level and albumin-creatinine ration between type 2 diabetic patients and normal controls. The age of type 2 diabetes mellitus was higher than normal controls (p<0.05). However, there was no significant difference in the age of different groups of diabetic patients.

**Table 1:** Clinical data of diabetic patients and normal controls.

Characteristics	Normal controls (n = 29)	Type 2 diabetes (total, n = 49)	Type 2 diabetes (total)			
			FPG		HbA1c%	
			GM1 (n = 25)	GM2 (n = 24)	BM1 (n = 25)	BM2 (n = 24)
Age(year)	50.93±9.94	60.00±11.80	60.68±10.34	59.29±13.35	59.60±12.65	60.42±11.11
Gender(m/f)	23/6	33/16	15/10	18/6	17/8	16/8
Duration(year)	/	7.15±7.03	6.45±7.71	8.43±6.55	6.89±5.86	7.56±8.81
FPG(mmol/l)	5.49±0.35	9.16±2.01	7.62±0.62	10.77±1.65	8.32±1.53	10.04±2.10
HbA1C%	5.16±0.34	7.70±1.26	7.27±1.09	8.14±1.30	6.73±0.42	8.70±1.04
HbA1c(mmol/mol)	32.85±3.71	60.61±13.82	55.94±11.91	65.47±14.22	50.07±5.58	71.59±11.41
CHOL(mmol/l)	5.04±0.78	5.13±1.17	4.96±1.25	5.32±1.08	5.02±1.33	5.25±0.99
TRIG(mmol/l)	1.19±0.48	1.53±0.83	1.64±1.04	1.43±0.51	1.46±0.65	1.62±0.99
HDL-C(mmol/l)	1.42±0.35	1.33±0.45	1.28±0.26	1.38±0.60	1.33±0.27	1.32±0.60
LDL-C(mmol/l)	2.98±0.65	3.06±1.01	2.83±0.98	3.30±1.01	2.93±1.05	3.19±0.97
Alb/Cr(mg/g)	<30	<30	<30	<30	<30	<30

Numbers are presented as mean ±SD unless otherwise indicated. HbA1c, haemoglobin A1c; FPG, fasting plasma glucose; HDL, high density lipoprotein; LDL, low density lipoprotein; TRIG, triglyceride; Alb/Cr, albumin/creatinine.

### Urine samples collection and preparation

Random midstream urine samples were collected in the morning into sterile polypropylene tubes from normal controls and type 2 diabetes mellitus. Immediately after collection, urine samples were centrifuged at 400 x g for 5min to remove cell debris and casts. Then supernatants were divided in aliquots and frozen at -80°C refrigeration until use. Prior to urine peptides isolation, urine samples were thawed at 4°C followed by centrifugation at 3000rpm for 10min, and then supernatants were immediately proceeded to peptides fractionation.

We used weak cationic-exchange magnetic beads to separate and purify urinary peptides through binding, washing, and elution according to the manufacturer’s instructions (Bruker Daltonics). First, 10ul MB-WCX and 95ul MB-WCX binding solution were added in a polypropylene tube, mixed thoroughly and then 10ul urine samples were added and mixed thoroughly. The tubes were placed in a magnetic bead separator (Bruker Daltonics) to separate the unbound solution. The magnetic beads were then washed three times. Second, 10ul MB-WCX eluting solution was added and mixed intensively by vortexing. Finally, the clear supernatant was transferred into a fresh tube, and 5ul MB-WCX stabilizing solution was added. The well-mixed eluate was then stored at—20°C.

### MALDI-TOF MS

The eluate of urinary sample was diluted 1:10 in matrix solution containing α-cyano-4-hydroxycinnamic acid (Bruker Daltonics). For example, 1μl of eluate was added to 10 μl matrix solution. Then 1ul of the resulting mixture was spotted onto the AnchorChip target (Bruker Daltonics), allowed to air dry and ionized by a nitrogen laser (λ = 337 nm) operating at 25 Hz. MALDI-TOF MS was performed using an Autoflex TOF instrument (Bruker Daltonics). Before data acquisition of every eight samples, the standard preparation would be calibrated. Eleven peptides were used as external standard preparation. Then, mass calibration was performed. For each MALDI spot, 400 spectra were acquired in analysis (50 laser shot at 8 different spot positions) and the average of 8 spots represents one urine sample.

### Data Processing

The spectra of all signals with a signal-to-noise ratio >5 were collected in the mass range of 1000–10000Da. All the spectra were analyzed using ClinProTools2.1 software to normalize spectra (using total ion count), subtract baseline and determine peak m/z values and intensities. To align the spectra, a mass shift of no more than 0.1% was determined. The peak area was used as quantitative standardization. ClinProTools2.1 bioinformatics software was used to find out the differently expressed urinary peptides. Comparative analysis was carried out two times, one among GM1, GM2 and normal controls and the other among BM1, BM2 and normal controls. The comparison of the m/z values among three groups was performed by nonparametric tests: the Mann-Whitney U test (for binary comparisons) and the Kruskal–Wallis test (for multi-group comparisons) using ClinProTools2.1 bioinformatics software. In all cases two-tailed p<0.05 was accepted as statistically significant.

Receiver operating characteristic (ROC) curve analysis and area under the curve (AUC) calculations were performed directly with SPSS17.0 software to determine diagnostic efficacy of each single marker.

### Peptide identification

Nano-liquid chromatography-tandem mass spectrometry, which consisted of an Aquity UPLC system (Waters) and a LTQ Obitrap XL mass spectrometer (Thermo Fisher) equipped with a nano-ESI source, was used to identify the sequences of differential expression peptides. Firstly the peptide solutions were loaded to a C18 trap column (symmetry 180um×20mm×5um, nano Acquity) with the flow rate of 15ul/min for 3 min. Then the desalted peptides were analyzed by C18 analytical column (symmetry 75um×150um×3.5um, nano Acquity) at a flow rate of 400nl/min. The mobile phases A (5% acetonitrile, 0.1% formic acid, Sigma-Aldrich) and B (95% acetonitrile, 0.1% formic acid) were used for analytical columns. Gradient elution profile was as follows: 5%B from initial to 40min, 45%B from 40 min to 41min, 80%B from 41min to 45min, 80%B from 45min to 45.5min, 5%B from 45.5min to 60min, and 5%B in 60min. The MS instrument was operated in a data-dependent model. The range of full scan was 400–2000 m/z with a mass resolution of 100,000 (m/z 400). The ten most intense monoisotope ions were the precursors for collision induced dissociation for two consecutive scans per precursor ion followed by 90s of dynamic exclusion.

### Bioinformatics analysis

The obtained spectra were analyzed with BioworksBrowser3.3.1 SP1 (Thermo Fisher) and the information were matched against the IPI Human database (v3.45) using Sequest search. Parameters were set as follows: Delton≥0.1; Charge2+, Xcorr2.0; charge3+, Xcorr2.5; peptide probability< = 1e-003; parent ion masses tolerance: 50ppm; fragment ion masses tolerance: 1Da; enzyme: no enzyme; variable modification: oxidation of methionine.

## Results

### Urine peptidome and data analysis of type 2 diabetes patients and normal controls based on FPG

**Fig 1:**
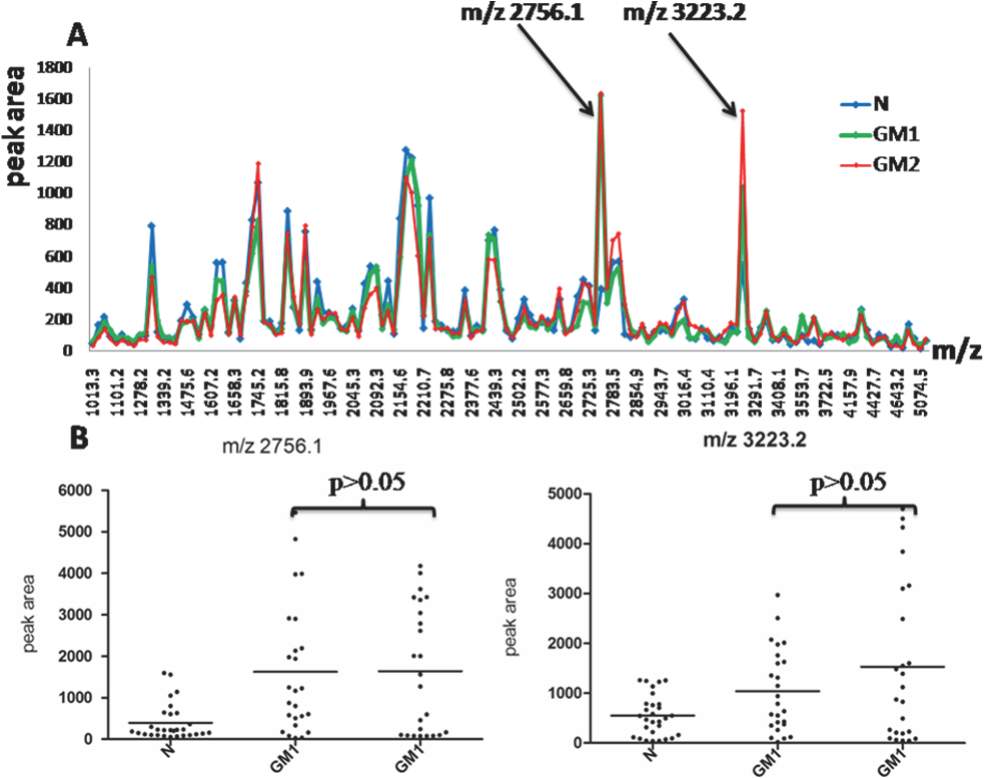
Analysis and comparison of urinary peptide peaks based on fasting plasma glucose of type 2 diabetes mellitus. (A) The distribution of the average peak area from the three groups and arrows indicating peaks that had statistically significant difference among three groups (p<0.05). (B) The peak area distributions of m/z 2756.1and m/z 3223.2 in all samples. Throughout comparison between two groups of the three, m/z 2756.1 and m/z 3223.2 had no significant difference (p>0.05) between GM1 and GM2.

Following the grouping as described in methods, the average intensity of 145 peaks that were detected by ClinprotTools2.1 available in the 1,000 to 10,000 m/z range is shown in Fig 1A. Arrows indicate peaks that the difference was statistically significant between the three groups and their distributions in all samples of the three groups are shown in Fig 1B. But, the intensity of peak m/z 2756.1 was not statistically significant between GM1 and GM2 (1627.2±1579.4 versus 1639.7±1524.4, p>0.05).

### Urine peptidome and data analysis of type 2 diabetes patients and normal controls based on HbA1c

**Fig 2:**
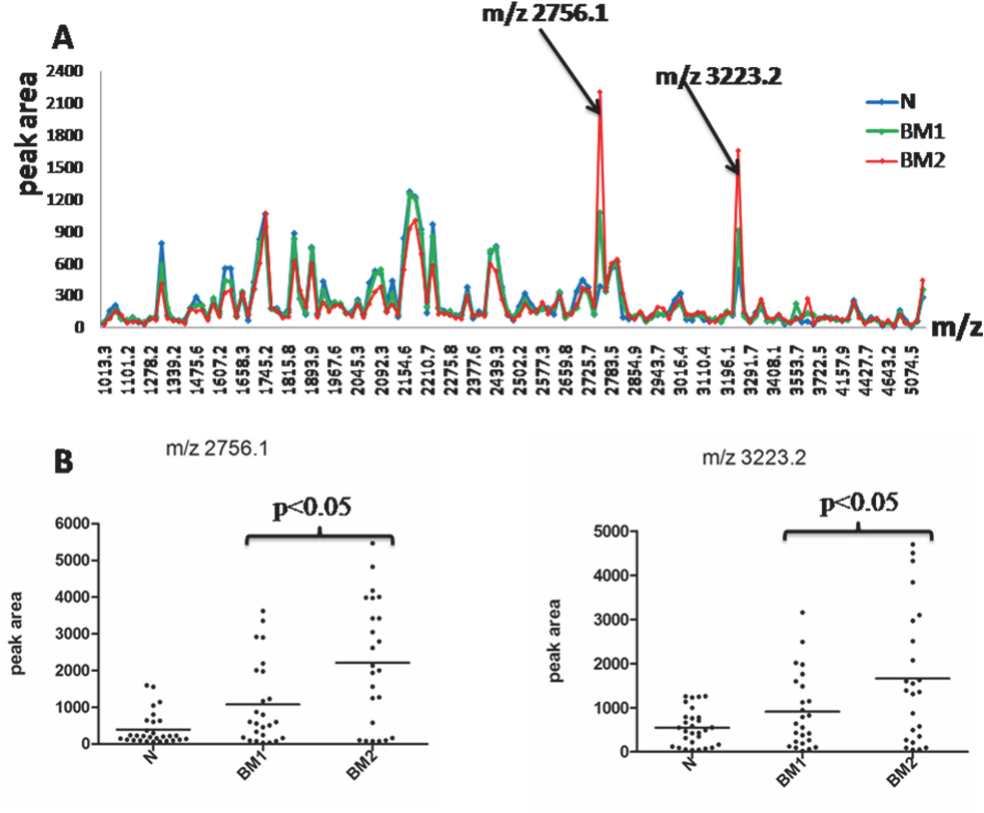
Analysis and comparison of urinary peptide peaks based on glycosylated haemoglobin A1c of type 2 diabetes mellitus. (A) The distribution of average peak area from the three groups and arrows indicate peaks that had significant difference among the three groups (p<0.05). (B) The peak area distributions of m/z 2756.1and m/z 3223.2 in all samples. Throughout comparison between two groups of the three, m/z 2756.1 and m/z 3223.2 had significant difference (p<0.05).

Following the grouping as described in methods, the average intensity of 145 peaks that were detected by ClinprotTools2.1 available in the 1,000 to 10,000 m/z range is shown in Fig 2A. We chose the peaks among the three groups in which the intensity was greater than 300 and the p value was less than 0.001. There were two peaks (m/z 2756.1and m/z 3223.2) that were differently expressed among the three groups (p<0.001) and the difference was also statistically significant between BM1 and BM2 (p<0.05). The distribution of the two highly expressed peaks in the three groups is shown in Fig 2B.

### Tendency analysis

**Fig 3:**
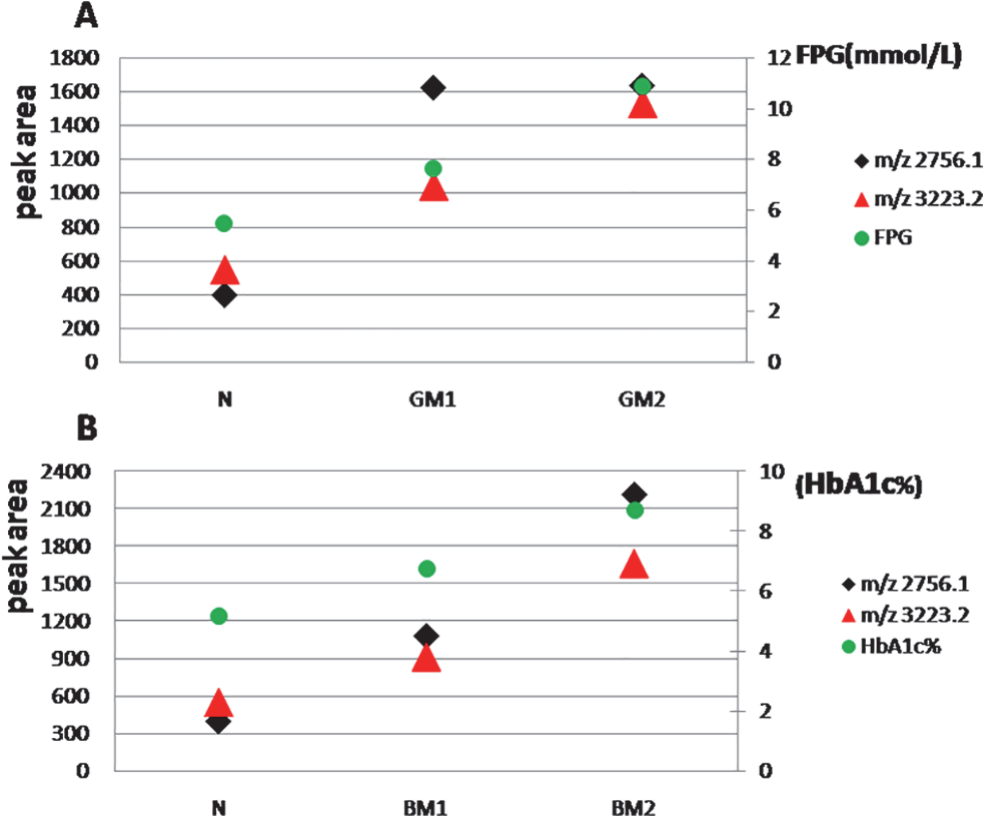
The changes trend between the differently expressed peaks and glycemic control. (A) Association between m/z 2756.1, m/z 3223.2 and fasting plasma glucose. (B) Association between m/z 2756.1, m/z 3223.2 and glycosylated haemoglobin A1c.

We analyzed the tendency between m/z 2756.1, m/z 3223.2 and fasting plasma glucose (Fig 3A). We also compared the two peaks with glycosylated haemoglobin and the parallelism between m/z 3223.2 and glycosylated hemoglobin was better than the parallelism between m/z 2756.1 and glycosylated hemoglobin (Fig 3B).

### ROC analysis

**Fig 4:**
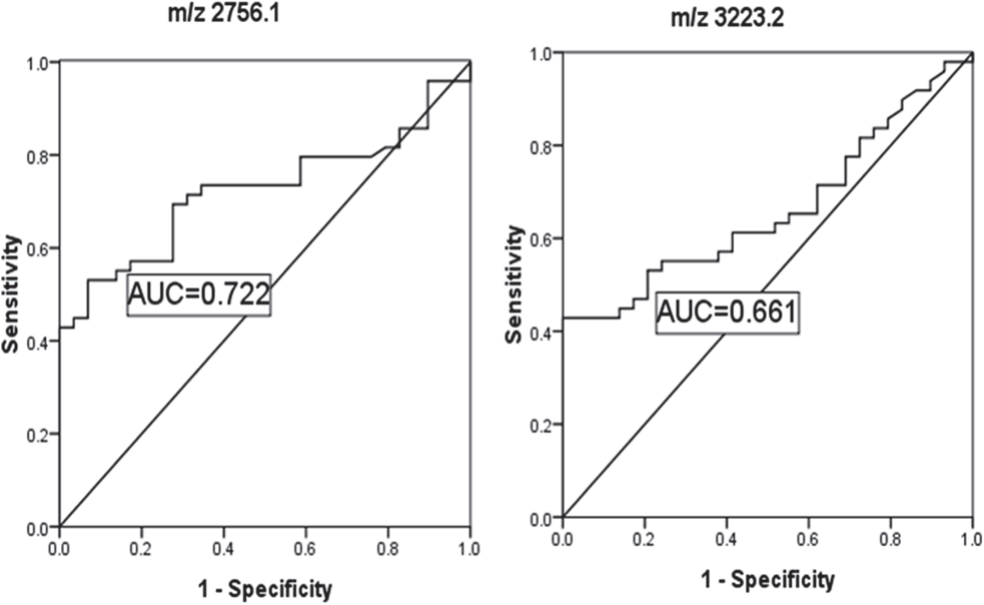
Receiver operated curve (ROC) and area under the curve (AUC) of the two urinary peptides based on glycosylated haemoglobin A1c of type 2 diabetes mellitus.

Receiver operating characteristic (ROC) curve and area under the curve (AUC) of the two identified peptides based on glycosylated haemoglobin are shown in Fig 4. AUC of the two peptides was 0.722 and 0.661, respectively.

### The identification of the two peptides

By nano-liquid chromatography–tandem mass spectrometry detection, the peptide sequences of the differential peaks were identified. The amino acid sequences of m/z 2756.1 were SYSKQFTSSTSYNRGDSTFESKSY and m/z 3223.2 GLRPLFEKKSLEDKTERELLESYIDGR. Through the Sequest search, the peptides of m/z 2756.1and 3223.2 were found to be partial sequences of fibrinogen alpha chain precursor and prothrombin precursor, respectively. The complete identification results are shown in Table 2.

**Table 2:** Identified peptides sequences of two elevated peaks.

m/z	Molecular weight	Amino sequences	Protein name and corresponding IPI
2756.1	2756.22	S.SYSKQFTSSTSYNRGDSTFESKSY.K	IPI00021885 Isoform 1 of Fibrinogen alpha chain precursor
3223.2	3220.74	C.GLRPLFEKKSLEDKTERELLESYIDGR.I	IPI00019568 Prothrombin precursor

## Discussion

Diabetes mellitus is currently one of the world’s fastest-growing diseases. In the daily self care of patients with type 2 diabetes, the most important thing is to monitor the change of blood glucose. Both clinical monitoring and self-monitoring of diabetes necessitate puncture blood, usually needing fasting at least eight hours, thus bringing great inconvenience to the patient. So, if we could find some noninvasive methods that associate with the level of plasma glucose and replace plasma testing, it would be a breakthrough for both clinicians and patients.

Urine has become one of the most attractive biofluids in clinical diagnostics as it can be obtained non-invasively in large quantities and is stable compared with other biofluids. Urine is the ultrafiltrate of blood and it is a window to reflect the actual changes of the body as some change of its composition may indicate certain disease states. Further, urinary peptides and lower molecular mass proteins are generally soluble and the protein content is relatively stable due to the fact that urine “stagnates” for hours in the bladder [12, 13]. Therefore, analysis of a urinary lower molecular mass peptide would be more helpful to diagnosis of disease and reveal pathophysiology of the disease. With the development of mass spectra technology, urinary proteomics has emerged as a promising tool for biomarker screening and identification. Bead based MALDI-TOF MS allows enrichment and analysis of the small proteins or peptides in different bio fluids, so the lower molecular mass compounds in the urine can now be analyzed in a mass spectrometer without additional manipulation (e.g. tryptic digests) [14].

In our study, MB-WCX was used to enrich the small proteins or peptides in the urine samples from 29 normal controls and 49 type 2 diabetes mellitus patients. Subsequently, MALDI-TOF MS was applied to screen the biomarker that is associated with different stages of plasma glucose control. First, we divided diabetes patients into two groups based on fasting plasma glucose. Compared with normal controls, Fig 1 shows the peaks m/z 2756.1 and m/z 3223.2 may be the differently expressed peptides, however, the statistical data analysis shows no difference between the two groups of type 2 diabetes mellitus patients. For all other proteins and peptides, the MALDI-TOF MS data analysis shows no statistically significant difference between the two groups of type 2 diabetes mellitus patients. Considering that glycosylated haemoglobin is more stable than fasting plasma glucose in the reflection of glucose control for diabetes mellitus patients, we divided the type 2 diabetes patients into two groups based on glycosylated haemoglobin. Fig 2A and Fig 2B show that m/z 2756.1 and 3223.2 were highly expressed in urine of type 2 diabetes patients compared with normal controls and that the peak intensity increased parallel to the increase of haemoglobin A1c in type 2 diabetes mellitus patients. Most importantly, the differences among the three groups are statistically significant and also in BM1 and BM2.

Pertinent to the identified peptides, a hypercoagulable state is defined as follows: patients have laboratory abnormalities or clinical conditions that are associated with increased risk of thrombosis [15]. In diabetes mellitus patients, a hypercoagulable state is associated with the increase in thrombosis and 80% of type 2 diabetic patients die from the thrombosis. Seventy-five percent of these deaths result from cardiovascular events and the remainder are due to cerebrovascular events and peripheral vascular complications [16]. An elevated level of coagulation factors suggested to contribute to hypercoagulability and is considered one of the risk factors that plays an important part in the development of stroke and myocardial infarction [17–19].

The two highly expressed peptides were identified as fragments of isoform 1 of fibrinogen alpha chain precursor and prothrombin precursor. Fibrinogen and prothrombin play important roles in blood clotting mechanism and prothrombin processed into thrombin under the action of prothrombin activator, and fibrinogen becomes fibrin monomer under the action of thrombin. The urinary peptides that we identified in our study are the fragments of the product from the above-mentioned processes. In type 2 diabetes mellitus patients, there are some major pathological conditions that could be responsible for the presence of diabetes-related biomarkers in urine: oxidative stress, low-grade inflammation and endothelial damage [20]. Diabetes patients also have lipid and protein metabolic disorders. Increased lipid synthesis further stimulates artery smooth muscle cell proliferation, leading to vascular basement membrane thickening and resulting in microcirculation disturbance. Microcirculation disturbance is easy to cause tissue ischemia, hypoxia and produce a large number of reactive oxygen species [21–23]. Reactive oxygen species could injure vascular intimal membrane and put the patients with type 2 diabetes in a pathological state of blood coagulation and fibrinolysis. On the other hand, chronic hyperglucemia could result in hyperglycosylation of multiple proteins [24, 25] and make them have abnormal functions. Under this state, the signals could induce the corresponding cell to produce more proteins [26]. The two elevated urinary peptides represent main clinical parameters for the evaluation of the diabetic status which may be used as indicators of risk for diabetic vascular complications.

Noninvasive urine collection in combination with the development of modern mass spectrometry has become one of the most attractive areas of study as mass spectrometry-based urinary peptide profiling plays an important role in clinical research. It could provide a new analysis insight for monitoring the plasma glucose of diabetes patients and some powerful indicators for prediction of diabetic complications. At present, the biomarkers that are identified with proteomics methods have not been applied in clinical diagnosis due to the high cost and the complex sample preparation and statistical processing. Therefore, efforts have to be made to explore new methods and bioinformatics tools about low abundance proteins and peptides, and there is a great need to validate these panels of biomarkers on larger cohorts of patients.
